# Immune responses associated with protection induced by chemoattenuated PfSPZ vaccine in malaria-naive Europeans

**DOI:** 10.1172/jci.insight.170210

**Published:** 2024-05-08

**Authors:** Yoanne D. Mouwenda, Simon P. Jochems, Vincent Van Unen, Madeleine Eunice Betouke Ongwe, Wouter A.A. de Steenhuijsen Piters, Koen A. Stam, Marguerite Massinga Loembe, Betty Kim Lee Sim, Meral Esen, Stephen L. Hoffman, Peter G. Kremsner, Rolf Fendel, Benjamin Mordmüller, Maria Yazdanbakhsh

**Affiliations:** 1 Centre de Recherches Médicales de Lambaréné, Lambaréné, Gabon.; 2 Department of Parasitology, Leiden University Medical Center (LUMC), Leiden, Netherlands.; 3 Department of Immunology, Leiden University Medical Center, Leiden, Netherlands.; 4 Centre National de la Recherche Scientifique et Technologique, Institut De Recherche En Écologie Tropical, Libreville, Gabon.; 5 Africa Society of Laboratory Medicine, Headquarters, Johannesburg, South Africa.; 6 Sanaria Inc., Rockville, Maryland, USA.; 7 Protein Potential LLC, Rockville, Maryland, USA.; 8 Institute of Tropical Medicine, University of Tübingen, Tübingen, Germany.; 9 German Center for Infection Research, Partner Site Tübingen, Tübingen, Germany.; 10 Cluster of Excellence EXC 2124, Controlling Microbes to Fight Infection, Tübingen, Germany.; 11 Radboud University Medical Center (Radboudumc), Department of Medical Microbiology, Nijmegen, Netherlands.

**Keywords:** Immunology, Vaccines, Cellular immune response, Cytokines, Malaria

## Abstract

Vaccination of malaria-naive volunteers with a high dose of *Plasmodium falciparum* sporozoites chemoattenuated by chloroquine (CQ) (PfSPZ-CVac [CQ]) has previously demonstrated full protection against controlled human malaria infection (CHMI). However, lower doses of PfSPZ-CVac [CQ] resulted in incomplete protection. This provides the opportunity to understand the immune mechanisms needed for better vaccine-induced protection by comparing individuals who were protected with those not protected. Using mass cytometry, we characterized immune cell composition and responses of malaria-naive European volunteers who received either lower doses of PfSPZ-CVac [CQ], resulting in 50% protection irrespective of the dose, or a placebo vaccination, with everyone becoming infected following CHMI. Clusters of CD4^+^ and γδ T cells associated with protection were identified, consistent with their known role in malaria immunity. Additionally, EMRA CD8^+^ T cells and CD56^+^CD8^+^ T cell clusters were associated with protection. In a cohort from a malaria-endemic area in Gabon, these CD8^+^ T cell clusters were also associated with parasitemia control in individuals with lifelong exposure to malaria. Upon stimulation with *P*. *falciparum*–infected erythrocytes, CD4^+^, γδ, and EMRA CD8^+^ T cells produced IFN-γ and/or TNF, indicating their ability to mediate responses that eliminate malaria parasites.

## Abstract 2

CD56^+^CD8^+^ T cells and EMRA CD8^+^ T cells associate with protection against malaria in both malaria-naive Europeans immunized with PfSPZ-CVac [CQ] and Africans with life-long exposure to malaria.

## Graphical abstract

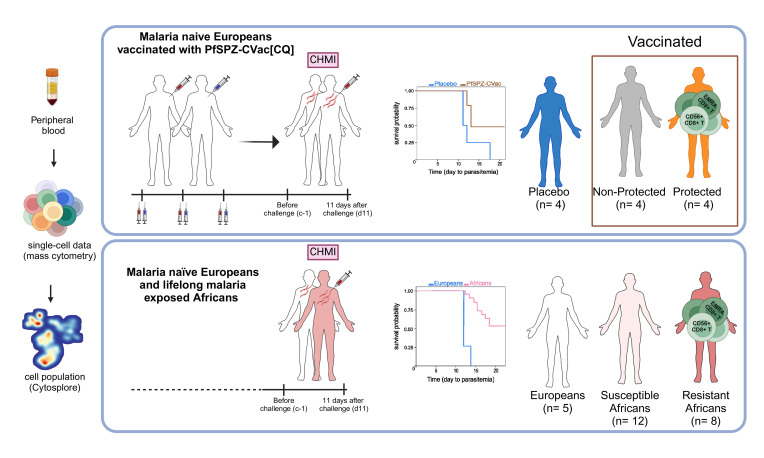

## Introduction

Despite the significant efforts and investments to complement the current control strategies with an effective vaccine (1), malaria remains a major global health problem, with 249 million cases and an estimated 608,000 deaths in 2022 (2). Although promising results have been reported with the R21/Matrix-M malaria vaccine (2, 3), until recently, the only malaria vaccine recommended by the WHO was RTS’S, which provides partial protection of between 18% and 28% over 4 years in young children without booster vaccination (4, 5). Recently, inoculation with *Plasmodium falciparum* sporozoites (SPZs) chemoattenuated with chloroquine (CQ) (PfSPZ-CVac [CQ]) was shown to induce dose-dependent protection that ranged between 33% and 100%, against *P*. *falciparum* after controlled human malaria infection (CHMI) with PfSPZ (6–8). Vaccine efficacy was associated with a significant increase in polyfunctional memory CD4^+^ T cells in response to SPZ or infected red blood cell antigens, as well as in circulating γδ T cells identified by flow cytometry (6), while the frequency of detectable antigen-specific CD8^+^ T cells was low (6). These results are consistent with previous flow cytometry studies that have linked human CD4^+^ and γδ T cells to the control of parasitemia in protected individuals, while the CD8^+^ T cells response could not be robustly captured (9, 10). However, studies in nonhuman primates vaccinated with attenuated SPZs have shown that, although circulating antigen-specific CD8^+^ T cells were present in the periphery, their frequency was much higher in the liver (11, 12). The development of mass cytometry provides the opportunity to study, in much greater detail, the specific immune cells associated with vaccine immunogenicity or efficacy (13, 14). In a recent study investigating the cellular immune profile and dynamic changes in immune responses to malaria parasites in preexposed Africans and malaria-naive Europeans, mass cytometry was capable of identifying novel immune signatures associated with naturally acquired immunity that controls parasitemia (15).

In the present study, we used mass cytometry (16) to characterize the immune profiles of malaria-naive Europeans vaccinated with PfSPZ-CVac [CQ] or placebo prior to CHMI, as well as the changes in immune responses after CHMI to identify immune profiles and function associated with protection at a much higher granularity compared with conventional flow cytometry. With this vaccination approach, we found that CD8^+^ T cell responses can be induced, detectable in human peripheral blood, and associate with vaccine-induced protection.

## Results

### Study participants.

**Figure 1:**
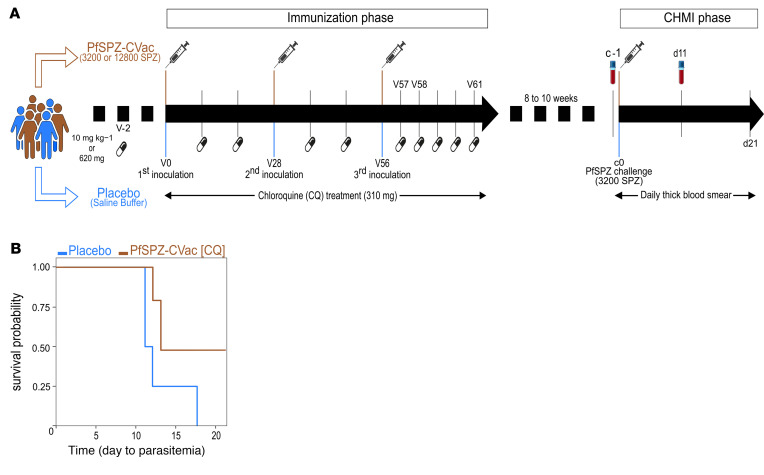
TüCHMI trial and outcome. (**A**) Healthy volunteers included in the trial were split in 2 main groups: the experimental group (in brown) consisted of volunteers (*n* = 8) receiving 3 doses of PfSPZ vaccine at 28-days intervals (V0, V28, and V56) in combination with a weekly dose of chloroquine up to 5 days after the last inoculation (V61) (PfSPZ-CVac [CQ]), and the placebo group (in blue), which consisted of volunteers (*n* = 4) inoculated with saline buffer. Eight to 10 weeks after the last inoculation, all volunteers in both the experimental and placebo groups underwent a CHMI trial. Immune responses to PfSPZ-CVac [CQ] inoculation were assessed at c–1 (1 day before the challenge [c0]) and d11 (11 days after the challenge). (**B**) Proportion of protected volunteers. Kaplan-Meier survival curves for days to parasitemia determined by thick blood smear for PfSPZ-CVac [CQ]–vaccinated (brown) and placebo (blue) groups. Volunteers in the placebo group all became malaria positive by day 18 after CHMI, while in the vaccinated group, some volunteers (4 of 8 volunteers vaccinated in total) remained malaria negative up to 21 days after CHMI.

Of the 12 volunteers studied, 4 received saline (placebo group) and 8 were vaccinated 3 times with PfSPZ-CVac [CQ] at 28-day intervals (6). To assess the ability of PfSPZ-CVac [CQ] to induce protective immunity, all 12 volunteers underwent CHMI by direct venous inoculation of live PfSPZ (6). All 4 volunteers in the placebo group developed parasitemia following CHMI, as indicated by thick blood smear (TBS) and PCR data (Supplemental Table 4; supplemental material available online with this article; https://doi.org/10.1172/jci.insight.170210DS1). Four of 8 volunteers were vaccinated with 3 doses of 3,200 SPZ and the rest with 12,800 SPZ. Irrespective of the vaccine dose, 4 of 8 volunteers remained thick blood smear (TBS) and PCR negative throughout the 21-day follow-up period after CHMI and were referred to as protected (Supplemental Table 4). The remaining 4 vaccinated volunteers, developed parasitaemia and were referred to as nonprotected (Figure 1B and Supplemental Table 4). Four volunteers (2 per vaccine dose) developed parasitemia (referred to as the nonprotected group) (Figure 1B and Supplemental Table 4). Baseline demographic data for the volunteers are shown in Table 1. Groups were similar with regard to age, sex, and BMI.

### Vaccine-induced immune cells detected prior to CHMI that associate with protection against malaria.

We investigated the immune response to PfSPZ-CVac [CQ] associated with protection at the pre-CHMI time point (c–1; 8–10 weeks after the third PfSPZ-CVac [CQ] vaccination and 1 day prior to CHMI). Unsupervised clustering using hierarchical stochastic neighbor embedding (HSNE) (17, 18) identified a total of 103 distinct cell clusters (Supplemental Figure 1), classified into lineages and subsets (Supplemental Table 1) based on their marker expression. Seven major immune lineages were annotated: CD4^+^ T cells, CD8^+^ T cells, γδ T cells, unconventional T cells, B cells, and innate lymphoid cells (including NK cells) as well as monocytes and DCs (Supplemental Figure 1).

**Figure 2:**
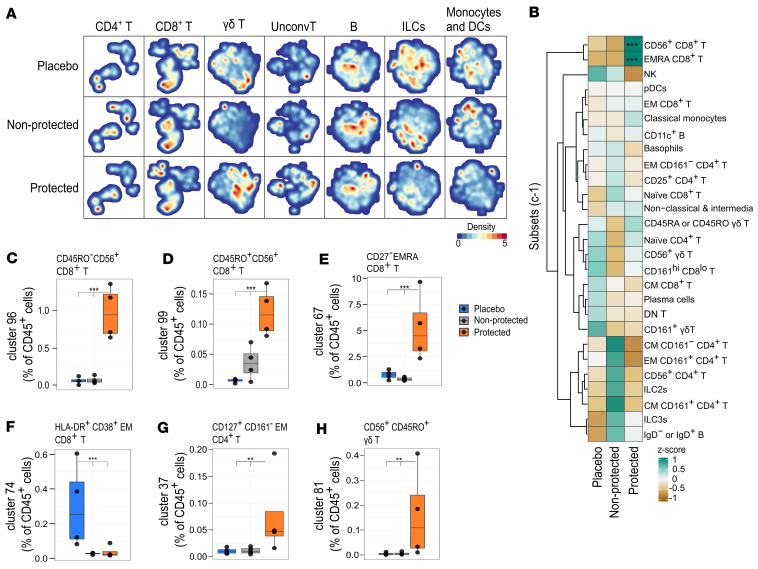
Vaccine-induced immunity associated with protection prior to CHMI. (**A**) Hierarchical stochastic neighbor embedding density maps showing differences in major cell lineages among volunteers n the placebo (*n* = 4), nonprotected (*n* = 4), and protected (*n* = 4) groups. The cell density per individual map is indicated by color. (**B**) Heatmap summary of *Z* scores of the normalized cell count per cell subset per group, where colors represent the mean *Z* score as indicated. (**C**) Box plots showing the frequency of CD56^+^CD8^+^ T cell cluster 96, (**D**) CD56^+^CD8^+^ T cell cluster 99, (**E**) EMRA CD8^+^ T cell cluster 67, (**F**) HLA-DR^+^CD38^+^ EM CD8^+^ T cells, (**G**) CD4^+^ T cell cluster 37, and (**H**) CD56^+^γδ T cell cluster 81 relative to CD45^+^ cells, comparing placebo (*n* = 4, blue), nonprotected (*n* = 4, gray), and protected (*n* = 4, orange) groups. The box plots represent the median and first and third quantile, and the whiskers represent the maximum/minimum, no further than 1.5 times the interquartile range (IQR). *P ≤ 0.05, **P < 0.01, ***P < 0.001, computed using the GLME model after FDR correction.

The difference in cell frequency prior to CHMI among the placebo, nonprotected, and protected groups was calculated using generalized linear mixed-effects (GLME) model (FDR ≤ 0.05), controlled for within-individual variation. Although distinct patterns were seen in the distribution of cells, as visualized in the HSNE map among the placebo, nonprotected, and protected groups (Figure 2A), there were no statistical differences in the percentage of cells at the lineage level among the groups prior to CHMI (Supplemental Figure 2A). At the subset level, only the frequency of terminally differentiated effector memory cells reexpressing CD45RA, EMRA (CD45RA^+^CCR7^–^) CD8^+^, and CD56^+^CD8^+^ T cell subsets were increased significantly in the protected group compared with the nonprotected and placebo groups (Figure 2B and Supplemental Figure 2, B and C). A more detailed examination of the CD56^+^CD8^+^ T cell subset revealed that clusters 96 and 99 displayed differential expression of CD45RO (Supplemental Figure 1B). In addition, unlike many other CD56^+^CD8^+^ T cell clusters, these clusters lacked the expression of CD161, CD27, or CD127 (Supplemental Figure 1B). Thus, these cell clusters accounted for the association of the CD56^+^CD8^+^ T cell subset with protection as tested by CHMI (Figure 2, C and D).

Cluster 67 of EMRA CD8^+^ T cells, distinguished by its lack of CD27 expression (Supplemental Figure 1B), was also significantly more abundant in the protected group compared with the nonprotected and placebo groups (Figure 2E). In addition, we found that cluster 74 of EM CD8^+^ T cells expressing HLA-DR and CD38 (Supplemental Figure 1B) were significantly increased in the placebo group compared with the vaccinated groups (Figure 2F).

Moreover, we characterized in more depth, CD4^+^ T cells and γδ T cells that Mordmüller et al. (6) identified to be associated with protection. We observed that the frequency of the EM CD4^+^ T cell cluster expressing PD1 (cluster 37) but negative for CD161 and CD28 (Supplemental Figure 1A) and γδ T cell cluster 81 expressing CD56, CD45RO, and CD11c (Supplemental Figure 1C) was significantly higher in the protected groups compared with the nonprotected and the placebo groups (Figure 2, G and H).

These results show, at a high resolution, which clusters of EM CD4^+^ T cells and memory γδ T cells, that were found by conventional flow cytometry (6), are associated with protection against malaria. Importantly, we also detected distinct CD8^+^ T cell populations in peripheral blood that were induced by vaccination with PfSPZ-CVac [CQ] and were associated with protection against malaria.

### Dynamic changes of immune cell clusters following CHMI.

Next, the changes over time following PfSPZ challenge in the 3 groups (placebo, nonprotected, and protected) were examined.

The change in cell frequencies per group between time points before CHMI (c–1) and 11 days after CHMI (d11) were assessed using the GLME model (FDR ≤ 0.05), including time as fixed effect and sample ID as random effect. Within each of the groups, we observed variations in the frequency of cell clusters over time. Notably, the placebo group exhibited more pronounced changes, potentially linked to their initial exposure to malaria parasites. In contrast, the observed changes in the nonprotected and protected groups may reflect cumulative effects stemming from the vaccinations and the associated protection (or lack thereof) (Supplemental Table 5). However, it is important to acknowledge that these results did not consider the potential confounding by immune profiles prior to vaccination.

**Figure 3:**
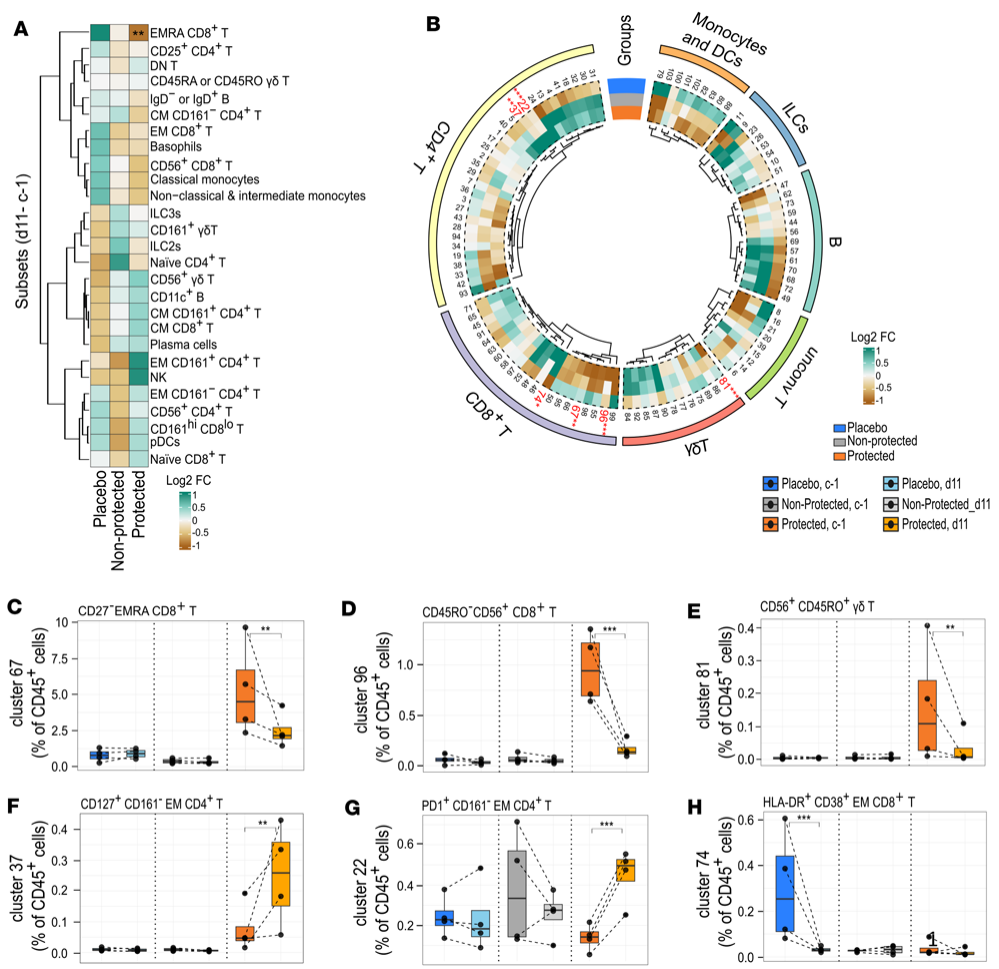
Dynamic changes of immune cell clusters following CHMI. (**A**) Heatmap summary of log_2_ fold change (FC) of cell subsets from c–1 to d11. (**B**) Circos heatmap showing the log_2_ FC from c–1 to d11 for each cluster per group. Clusters that significantly change overtime are in red. (**C**) The frequency of EMRA CD8^+^ T cell cluster 67, (**D**) CD56^+^CD8^+^ T cell cluster 96, (**E**) CD56^+^ γδ T cell cluster 81, (**F**) CD161^–^ EM CD4^+^ T cell cluster 37, (**G**) CD161^+^ EM CD4^+^ T cell cluster 22, and (**H**) HLA-DR^+^CD38^+^ EM CD8^+^ T cells (cluster 74), from c–1 to d11 per placebo (*n* = 4, blue), nonprotected (*n* = 4, gray), and protected (*n* = 4, orange) groups. Data in **C**–**H** are presented as box plots, representing the median and first and third quantile, while the whiskers indicate the overall data range no further than 1.5 times the interquartile range (IQR). The interaction among the groups and the time points was computed in a generalized linear mixed models (GLMM) for binomial family) model to assess the dynamic change overtime. *P ≤ 0.05, **P < 0.01, ***P < 0.001 after FDR correction. The abundance of the indicated clusters is given as a percentage of CD45^+^ cells.

To address this, we incorporated an interaction term among the groups and the CHMI time points (c–1 and d11) in our analysis. This approach allowed us to statistically test whether changes over time are significantly different among the groups, thus identifying immune cell populations associated with protection with respect to placebo. At the lineage level, no differences in cell frequencies were seen between groups. However, within the protected group, the frequency of EMRA CD8^+^ T cell subset decreased significantly over time (Figure 3A). This was further illustrated at the cluster level by the significant decrease at d11 of the frequency of EMRA CD8^+^ T cell cluster 67, which was seen to be elevated at the prechallenge time point in the protected group (Figure 3, B and C).

Similarly, a decrease was observed in CD56^+^CD8^+^ T cell cluster 96, which again was significantly higher at prechallenge (Figure 3, B and D). This may reflect that upon exposure the reactive cells induced by vaccination relocate from the periphery to tissues, such as the liver, where they may play a protective role against malaria. Alternatively, these cells can become activated or die. In addition, we show that, in the protected group, the frequency of γδ T cells expressing CD56, CD45RO, and CD11c (cluster 81), which was significantly elevated prechallenge, decreased significantly (Figure 3, B, E, and F). In contrast, the frequency of CD161^–^ EM CD4^+^ T cells expressing PD1 (cluster 37), which was also higher at prechallenge, increased significantly. A new cluster emerging from this analysis, the CD161^+^ EM CD4^+^ T cells (cluster 22) (Supplemental Figure 1A), which has been shown to be associated with protection in individuals with naturally acquired immunity in endemic areas (15), increased significantly in the protected group (Figure 3G). Only one cell cluster differentially changed in the placebo group after CHMI (Figure 3B); the frequency of EM CD8^+^ T cells expressing HLA-DR and CD38 (cluster 74), which was high at the prechallenge time point in the placebo group, decreased significantly at d11 (Figure 3H). The cell abundance of cluster 74 at d11 in the placebo group is similar to that observed at c–1 in the protected and the nonprotected groups (Figure 3H), which might suggest that these cells respond specifically to encountering PfSPZ inoculum.

Overall, these results suggest that upon challenge the CD56^+^ and EMRA CD8^+^ T cell clusters, which we were able to detect using mass cytometry, leave the peripheral blood, as do γδ T cells, possibly to mediate protection against malaria parasites in the liver. At the same time, CD4^+^ T cells increase over time in the protected group, confirming earlier reports of their role in immunity.

### Comparison of CD8^+^ T cells in naturally acquired and vaccine-induced immunity.

Given the current detection and characterization of CD8^+^ T cell responses in vaccine-induced immunity to malaria, we next asked whether we could find similar CD8^+^ T cell clusters in naturally acquired immunity in people with lifelong exposure to malaria.

**Figure 4:**
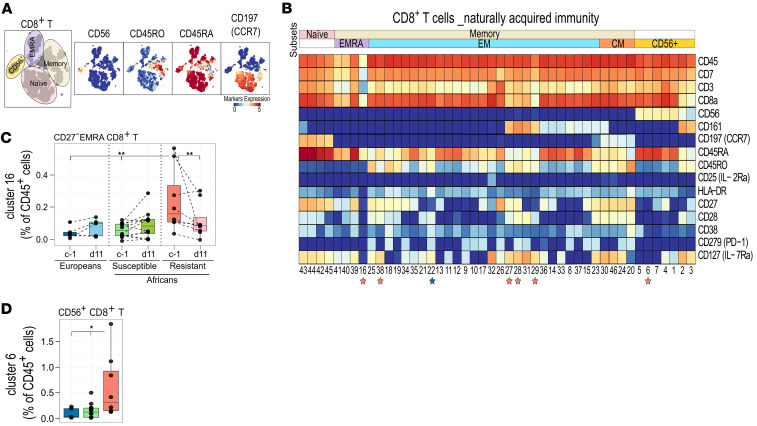
Characterization of CD8^+^ T cells in naturally acquired immunity. (**A**) HSNE plots showing cell subsets within the CD8^+^ T cells lineage (left), annotated based on the indicated markers (right). Colors represent the arc-hyperbolic sine 5–transformed (arsinh-5–transformed) marker expression as indicated. (**B**) Heatmap of CD8^+^ T cell clusters, showing expression of markers as median signal intensity after arsinh transformation. Each cluster has a unique cluster number, and the subset to which each cluster belongs is shown at the top. The generalized linear mixed models (GLMM) for binomial family was used to compare cluster abundance among malaria-naive Europeans (*n* = 5, blue), lifelong exposed susceptible Africans (*n* = 12, green), and resistant Africans (*n* = 8, pink). Colored stars below the clusters indicate statistical significance in naive Europeans and lifelong exposed resistant Africans. (**C** and **D**) Box plot representing the median and first and third quantile of the frequency of (**C**) EMRA CD8^+^ T cells (cluster 16) and (**D**) CD56^+^CD8^+^ T cells (cluster 6), both relative to CD45^+^ cells. The whiskers of the box plots indicate a range no further than 1.5 times the interquartile range (IQR). *P ≤ 0.05, **P < 0.01, ***P < 0.001. *P* values were computed using the generalized linear mixed models (GLMM) for binomial family.

To this end, we used a data set generated in a CHMI study in Gabon (LaCHMI-001 trial; ClinicalTrials.gov NCT02237586) (19), which identified individuals with naturally acquired immunity that controlled parasitemia, using mass cytometry panel with an identical set of markers as those used in our panel and applied our analysis pipeline (15). In this study, a total of 20 individuals from Gabon with prior malaria exposure and 5 malaria-naive Europeans underwent a CHMI (15). All malaria-naive individuals subsequently tested positive for malaria infection. Within the group with prior malaria exposure, 12 of 20 participants developed parasitemia as determined by TBS, while 8 participants did not develop parasitemia up to 28 days after the CHMI, when they received presumptive treatment. The frequencies of cell clusters prior to CHMI (c–1) and following PfSPZ challenge (at d11) were assessed using generalized linear mixed models (*P* < 0.05), comparing malaria-naive Europeans, preexposed susceptible Africans (Africans who develop parasitemia within 28 days after the CHMI) and preexposed resistant Africans (Africans who did not develop parasitemia up to 28 days after the CHMI). Using unsupervised clustering with HSNE in Cytosplore, CD56^+^, EMRA, EM, CM, and naive subsets were identified within the CD8^+^ T cell compartment (Figure 4A). The frequency of these subsets did not differ significantly among the groups. However, at the cluster level, 7 clusters of CD8^+^ T cells were significantly different among the groups prior to CHMI (Figure 4B).

Interestingly, a cluster of EMRA CD8^+^ T cells (cluster 16) was strongly associated with resistance as assessed after CHMI (Figure 4B) and found to significantly decreased over time in preexposed Africans (Figure 4C). This is a cluster of EMRA cells phenotypically similar to cluster 67 of EMRA CD8^+^ T cells (Supplemental Figure 1B), which as described above in our vaccine trial were high prior to CHMI and significantly decreased at d11 in PfSPZ-CVac [CQ] protected vaccinees (Figure 3C). Similarly, cluster 6 of CD56^+^CD8^+^ T cells (Figure 4B) was found to be high in the resistant group at c–1 (Figure 4D), which is in line with the finding of our trial that cluster 96 of CD56^+^CD8^+^ T cells (Supplemental Figure 1B) was high prior to CHMI in the vaccinated group that were protected, compared with other groups (Figure 2C). However, no significant decrease was seen at d11 for cluster 6 (Supplemental Figure 2D). Taken together, these data indicate that similar clusters of CD8^+^ T cells are associated with both naturally acquired and PfSPZ-CVac [CQ]–induced immunity.

### Functional responses associated with control of parasitemia.

We next assessed functional responses by measuring intracellular expression of cytokines in response to *P*. *falciparum*–infected red blood cells (PfRBCs) in CD4^+^, CD8^+^, and γδ T cells, which were found to be associated with PfSPZ-CVac [CQ]–induced protection. Specifically, while antigen-specific robust IFN-γ and TNF responses were measured following PfRBC stimulation (Supplemental Figure 3A), compared with uninfected red blood cells (uRBCs) as control (Supplemental Figure 3B), other cytokines (IL-2, IL-4, IL-5, IL-13, IL-17, and IL-10) were produced in negligible amounts.

**Figure 5:**
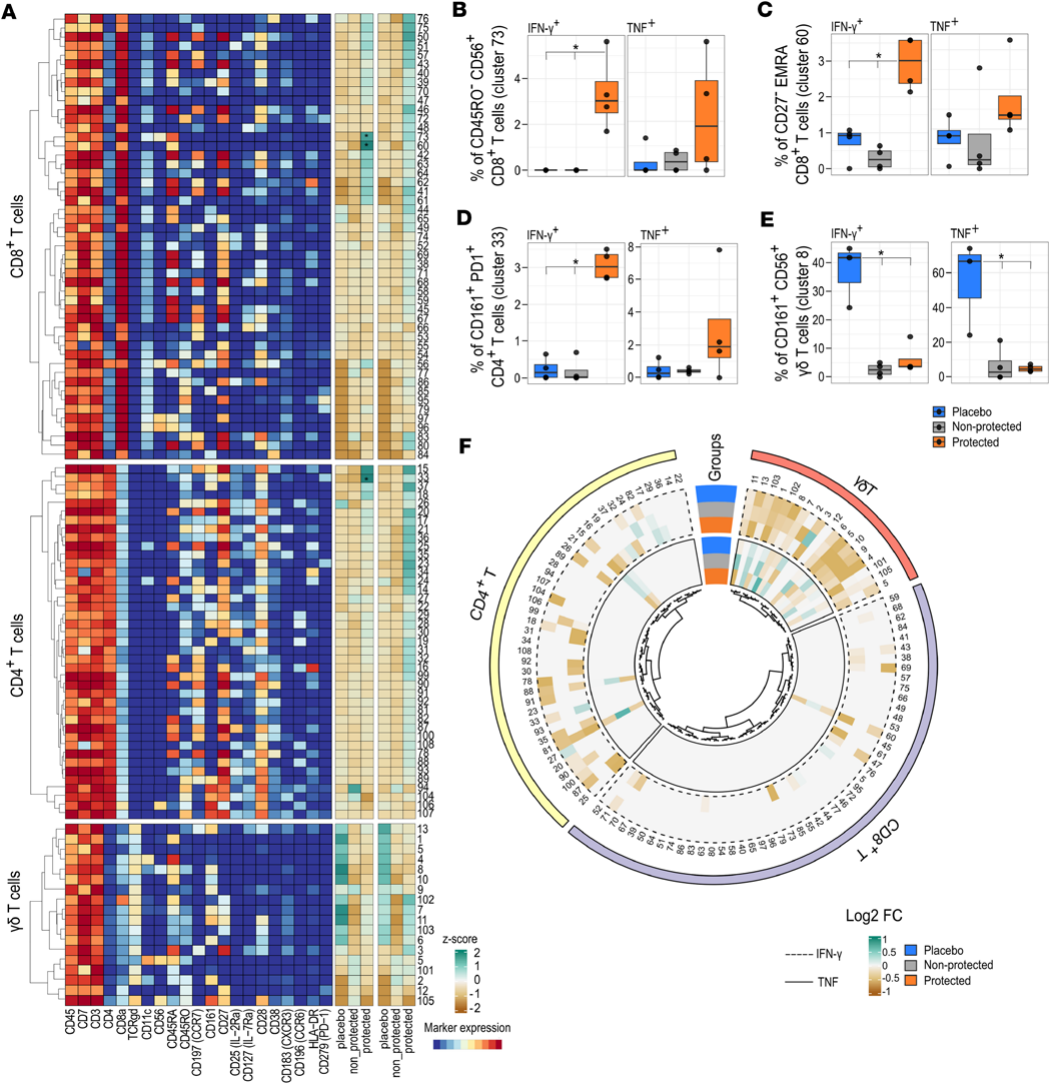
Cytokine response to PfRBC stimulation. (**A**) The heatmap on the left shows the expression of markers as median signal intensity after arsinh transformation for CD8^+^, CD4^+^, and γδ T cells. Each cluster has a unique number. The heatmap on the right shows the summary of *Z* scores of the normalized frequency of cells producing IFN-γ and TNF per cluster (after subtraction of uRBCs) in placebo (*n* = 4), nonprotected (*n* = 4), and protected (*n* = 4) groups at baseline. The colors indicated represent the mean *Z* score per cluster per group. (**B**) Frequency of IFN-γ– and TNF-producing CD56^+^CD8^+^ T cells (cluster 73), (**C**) EMRA CD8^+^ T cells (cluster 60), (**D**) CD161^+^ EM CD4^+^ T cells, and (**E**) CD56^+^ γδ T cells, given as a percentage of parents among the indicated groups. The data are presented as box plots showing the median, the first, and the third quantile, and whiskers extend to the maximum/minimum, no further than 1.5 times the interquartile range (IQR). *P ≤ 0.05, computed using the generalized linear mixed models (GLMM) for binomial family. (**F**) Circos heatmap showing the log_2_ FC of IFN-γ– (dashed line) and TNF-producing (full line) cells from c–1 to d11.

Prior to CHMI, we observed, within the CD8^+^ T cell compartment, a significant increase in IFN-γ–producing (but not TNF-producing) CD56^+^CD8^+^ T cells (cluster 73) as well as EMRA CD8^+^ T cells (cluster 60) in the protected group (Figure 5, A–C). This indicates that EMRA cells induced by PfSPZ-CVac [CQ] are not hyporeactive and are able to respond to PfRBCs. Furthermore, EM CD4^+^ T cells also responded to PfRBC stimulation. Indeed, a cluster of EM CD4^+^ T cells expressing CD161, CD28, and PD1 (cluster 33) producing IFN-γ was found in higher frequency in the protected group prior to CHMI (Figure 5, A and D). Similarly, an increased frequency of IFN-γ– and TNF-producing CD56^+^ γδ T cells (cluster 8) expressing CD161 and CD45RO (Figure 5, A and E) was seen following PfRBC stimulation. However, a higher frequency of cytokine-producing γδ T cells was found in the placebo group compared with the nonprotected and protected groups.

A number of changes in cytokine-producing cell frequencies between c–1 and d11 (Figure 5F) that were not statistically significant following FDR correction might be worth considering. For example, we could see that the proinflammatory cytokine-producing CD56^+^ (cluster 73) and EMRA CD8^+^ T cells (cluster 60) decreased after PfSPZ challenge (Figure 5F and Supplemental Figure 3, C and D), in line with the decrease over time in the protected group of CD56^+^ and EMRA CD8^+^ T cell frequencies, as seen in Figure 3, C and D, suggesting the relocation of such cells to the liver. Interestingly, the frequency of CD56^+^ γδ T cells (cluster 8) producing proinflammatory cytokine was also seen to decrease at d11 (Figure 5F and Supplemental Figure 3E). This decrease could potentially be attributed to the relocation of these cells to the liver, resulting in reduced frequency of cytokine-producing cells. This observation aligns with the declining frequency of these cells from c–1 to d11. This also agrees with previous observations in endemic regions, where repeated malaria exposure has been shown to result in decreased frequency of proinflammatory cytokine-producing γδ T cells to antigen reexposure.

## Discussion

Using high-dimensional immune profiling, we could detect clusters of CD56^+^ and EMRA CD8^+^ T cells associated with PfSPZ-CVac [CQ]–induced immunity to malaria. In addition, we identified clusters that might be responsible for the reported association of CD4^+^ and γδ T cells with vaccine-induced immunity to malaria (6). Specifically, with regard to CD4^+^ T cells, we found total CD161^+^ EM CD4^+^ T cells and IFN-γ producing CD161^+^CD4^+^ T cells to increase over time in the protected vaccinees. This is consistent with previous data showing their association with naturally acquired immunity, induced after repeated exposure to malaria parasites in endemic areas (15). In our study, vaccinees received 3 inoculations of live chemoattenuated PfSPZ prior to CHMI. This regimen resulted in repeated exposure to early blood-stage malaria parasites that start to resemble what is seen in natural infections, which might explain the expansion of IFN-γ–producing CD161^+^ EM CD4^+^ T cells that contribute to vaccine-induced immunity as well as to naturally acquired immunity. Considering γδ T cells, we found that the population associated with protection expressed CD56. NK-like γδ T cells have been described to be associated with clinical immunity against malaria as a result of multiple exposures (20, 21), and it was reported that this also leads to a decrease in their proinflammatory cytokine production (21). Therefore, in our study, the repeated exposure, inherent to PfSPZ-CVac [CQ], could explain (a) the higher induction of CD56^+^ γδ T cells associated with protection and (b) the decrease in the placebo group in the frequency of IFN-γ– or TNF-producing CD56^+^ γδ T cells in response to malaria antigens in vitro on d11 after CHMI.

The protective CD8^+^ T cell responses against malaria, induced by both irradiated SPZs and various subunit vaccines (22–24), have been found to be correlated with liver-stage immunity (25, 26). Here, we could identify specific cell clusters and report that CD56^+^CD8^+^ T cells and EMRA CD8^+^ T cells are associated with protection against malaria in both malaria-naive Europeans immunized with PfSPZ-CVac [CQ] and Africans with lifelong exposure to malaria. Interestingly, in both populations, the frequency of CD56^+^ and EMRA CD8^+^ T cells was high prior to CHMI and decreased over time in the vaccine-induced or naturally acquired protected group exposed to CHMI, suggesting the migration of cells to the liver, where CD8^+^ T cells can directly act against liver-stage parasites (9, 11, 27). In this regard, an important study has shown that CD8^+^ tissue-resident T cells obtained by fine needle biopsy of liver have counterparts in peripheral blood, showing transcriptional similarity (25), which might warrant linking the activity of some circulating CD8^+^ T cells to their activity in the liver.

The CD56^+^CD8^+^ T cells that we found associated with protection might represent NKT cells, although specific NKT cell markers are required for their definitive identification (28). NKT cells are thought to prevent the development of blood-stage malaria by inhibiting the proliferation of parasites in the hepatocytes (29). There is currently limited evidence suggesting that EMRA CD8^+^ T cells play a role in protection against malaria. However, they are induced by live yellow fever vaccine (YF-17D) (30, 31) and are possibly involved in protection induced by the tetravalent live attenuated dengue vaccination (TV003) (32). EMRA cells are suggested to derive from antigen-specific cells reexpressing CD45RA as an indicator of highly functional memory CD8^+^ T cells (30, 33), yet the loss of CD27 on EMRA cells has been associated with low expansion potential in response to antigen stimulation in vitro (33, 34). Here, for malaria, we report that the frequency of total CD27^–^ EMRA CD8^+^ T cells and the frequency of IFN-γ^+^CD27^–^ EMRA CD8^+^ T cells was high at c–1 in the protected group in contrast to their EM counterpart. These observations suggest that EMRA cells might be a terminally differentiated but fully functional effector T cell population in the protected group. The significant decrease in EMRA frequency observed at d11 in the protected group might indicate that these cells respond specifically to PfSPZ infection after reexposure. This observation is in line with a previous report indicating that EMRA CD8^+^ T cells retain epigenetic markers that promote rapid effector function (35). The remaining question is how these cells are formed and where they relocate after the challenge. Analysis of the immune response before any vaccination and analysis of the TCR clonotype may help develop understanding of the specific kinetics of these cells in response to malaria infection. In addition, despite the obvious drawbacks, the inclusion of hepatic fine needle aspirates (25) to investigate the relationship between circulating EMRA CD8^+^ T cells and their liver-resident counterparts may provide further insight into the protective role of EMRA CD8^+^ T cells in human malaria infection.

Furthermore, we found the EM CD8^+^ T cell cluster, positive for HLA-DR and CD38, to be significantly depleted in the vaccinated group compared with the placebo group. Interestingly, this contrasts with results of a subunit vaccine, AdCA (an adenovirus-vectored malaria vaccine expressing *P*. *falciparum* circumsporozoite protein [CSP] and apical membrane antigen-1 [AMA1]), which did not induce a sterile protection (24). The AdCA vaccination induced an increase in antigen-specific CD8^+^ T cells expressing CD38 and HLADR at day 22 after immunization compared with baseline (before immunization). In our study, the after immunization time is 8–10 weeks after the last inoculation, which might then allow these cells to migrate into the lymph nodes or tissues of the PfSPZ-CVac [CQ]–immunized volunteers.

It is important to acknowledge the limitations inherent to controlled human infection studies, notably the small sample size, which can affect the statistical analysis and generalizability of findings. Further studies in a larger number of individuals vaccinated with chemoattenuated malaria parasites are needed to confirm these findings, to unequivocally establish that the cell clusters identified here represent correlates of immunity or might mediate protection against malaria parasites. Another limitation of this study is that we have tested whole parasite antigen extract and do not have data on the specific antigens that drive the responses; again future studies could contribute to this knowledge gap. Moreover, it would be interesting to assess whether the changes in overall cellular response to malaria vaccine and challenge through CHMI are different from responses to other live attenuated vaccines to establish whether there are some general rules on how certain types of vaccine responses can be optimized. The absence of baseline data is also a limitation. However, the analysis of baseline data on previously published data of the same cohort showed no differences among the groups (6). Nevertheless, the inclusion of baseline samples (before vaccination) could have strengthened our conclusions regarding the effect of vaccination with PfSPZ-CVac on the development of specific immune responses associated with protection. Notwithstanding these limitations, this work highlights how combining high-dimensional single-cell analysis with vaccination and the controlled human infection has the potential to identify cell populations involved in protection against malaria.

## Methods

### Sex as a biological variant.

Both male and female individuals were included in the study. The sex ratio is provided in Table 1. The potential influence of sex on the observed outcomes was not accounted for in our analysis.

### Study population and sampling.

Samples for this study were collected as part of the TüCHMI-002 trial, a randomized, placebo-controlled, double-blind study, evaluating the inoculation of aseptic, purified, cryopreserved, nonirradiated PfSPZ by direct venous injection to malaria-naive, healthy adult volunteers from Germany, who were taking CQ as chemoprophylaxis against malaria (Sanaria PfSPZ-CVac [CQ]). Healthy malaria-naive patients, aged 18–45 years, were divided into 2 main groups: the control or placebo group and the experimental group. Within the experimental group, volunteers were divided according to the concentration of *P*. *falciparum* SPZs (PfSPZ) they received during the immunization phase: a low dose (3,200 SPZ) or a medium dose (12,800 SPZ). Volunteers received 3 PfSPZ immunization or saline buffer at 28-day intervals, in combination with a weekly dose of 5 mg/kg or 310 mg CQ for 5 days after the last immunization, after an initial dose of 10 mg/kg or 620 mg CQ 2 days before the first immunization. Eight to 10 weeks after the last immunization, all volunteers (including those in the placebo group) underwent a CHMI trial. The parasitemia following the challenge was evaluated daily, using both qPCR and TBS, starting from day 6 after challenge up to day 21. The outcomes for parasitemia for each donor can be found in Supplemental Table 4. A TBS slide was considered positive when two separate readers detected at least 2 parasites in 300 reading fields. In cases where discrepancies arose in parasitemia results between the two readers, a third reader was asked to assess the slide. If only 1 parasite-like structure was detected by the readers, more reading fields were assessed. The sample was declared negative in cases in which no other parasite could be found. Correlation between qPCR and TBS results was seen, as presented in Supplemental Table 4. Notably, within the protected group, all participants tested negative for *P*. *falciparum* infection in both qPCR and TBS. Samples used in this study were collected on c–1 and d11 for PBMCs isolation by density-gradient centrifugation and cryopreserved for subsequent immunophenotyping assays (Figure 1A).

To compare vaccine-induced immunity with naturally acquired immunity, we used publicly available data from a previous study conducted in Gabon (ImmPort accession SDY1734) (15, 19). This allowed us to analyze and compare specifically the CD8^+^ T cell immune responses in these 2 groups.

### Cell processing.

Cryopreserved PBMCs were thawed in RPMI medium supplemented with penicillin/streptomycin (RPMI^pen/strep^) and complemented at 50% with heat-inactivated (hi) FCS. After thawing, 1–2 million cells were aliquoted in a 5 mL Eppendorf tube for direct staining with the panel in Supplemental Table 2. The remaining cells were rested for 4 hours at 37^o^C in culture medium (RPMI^pen/strep^ + 10% hiFCS) at a concentration of 1 million cells/mL. All samples met the acceptance criteria of more than 80% viability after thawing and resting. Next, to assess response to malaria antigens, PfRBCs were used as source of antigen. The PfSPZ-CVac immunization procedure leads to the exposure of the immune system not only to SPZs, but also to infected hepatocytes and even early blood-stage parasites. PfSPZ-CVac immunization includes the inoculation of SPZs that invade liver cells subsequent to injection, where they differentiate and multiply for about 1 week, followed by release into the bloodstream and infection of erythrocytes in the form of merozoites. Therefore, 1–3 million of the rested cells per sample were stimulated for 24 hours with PfRBCs or uRBCs at a concentration of 1:1 at 37^o^C and 5% CO_2_. Brefeldin A was added to the cells 4 hours before the end of the culture. PfRBCs and mock-cultured uRBCs were obtained through purifying NF54 asexual-stage cultures using a MACS column (Miltenyi Biotech, 130-042-401) and cryopreserved in 15% glycerol/PBS. After stimulation, cells were transferred to a 5 mL Eppendorf tube for staining with the panel in Supplemental Table 3.

### Staining procedures.

Prior to staining, cells were incubated with 1 mL 500 μM Cell-ID intercalator-103Rh (Fluidigm, catalog 201103A; ×500 dilution) for 15 minutes at room temperature to identify dead cells. Subsequently, cells were washed with 2 mL staining buffer (DVS science, catalog 201068). Cells were next incubated for 10 minutes with 50 μL human TruStain FcX Fc-receptor blocking solution (Biolegend; ×10 dilution) at room temperature. Then, 50 μL of the surface antibody cocktail was added to the cells (Supplemental Tables 1 and 2) for 45 minutes at room temperature. After staining, cells were fixed with 1 mL of ×1 MaxPar Fix I buffer (Fluidigm, catalog 201065) for 20 minutes at room temperature. Stimulated cells were then stained a second time with 50 μL of an intracellular antibody cocktail (Supplemental Table 2) freshly prepared in permeabilization buffer (Fluidigm, catalog 201066) for 30 minutes at room temperature. After staining, cells were incubated overnight at 4°C with 1 mL 125 μM Cell-ID Intercalator-Ir (Fluidigm, catalog 201192A; ×1,000 dilution) in MaxPar Fix and Perm buffer (Fluidigm, catalog 201067).

### Mass cytometry (CyTOF) measurements.

Cells were acquired with the Helios mass cytometer (Fluidigm) at a concentration of 1 × 10^6^ cells/mL in Milli-Q water (Ultrapure water systems, PureLAB Ultra) complemented with 10% EQ Four Element Calibration Beads (Fluidigm, catalog 201078). In addition to the antibody panel detection channels (including intercalator channels), calibration bead (140Ce, 151Eu, 153Eu, 165Ho, and 175Lu) and contamination (133Cd, 138Ba, and 208Pb) channels were activated. After cell acquisition, data from the calibration bead data were used to normalize the signal fluctuations. The normalized FCS files were exported and analyzed with FlowJo V10 (TreeStar) to exclude EQ beads and select live CD45^+^ cells (Supplemental Figure 4A). The FCS files from selected live CD45^+^ cells were then analyzed using the hierarchical stochastic neighborhood embedding (HSNE) method on Cytosplore (17, 18), a dimensionality reduction visualization tool (Supplemental Figure 4B). The HSNE method of clustering enables the selection of cell landmarks (also referred to as clusters) per level based on the similarity of their marker expression. At the first level of the HSNE (overview level), major cell lineages were defined (i.e., CD8^+^ T cells). Next, we zoomed into each lineage separately to a more detailed level where we defined cell subsets (i.e., CD56^+^CD8^+^ T cells). The HSNE density plot allows the visualization of the distribution of the cells, indicating their similarity in the HSNE embedding, suggesting distinct immune signatures in a given immune compartment. The same process was repeated until the clusters level was reached (Supplemental Figure 4B). Each cluster represents a unique phenotype (Supplemental Figure 1). For each cluster, the percentage of IFN-γ– or TNF- producing cells was filtered using a manually determined cytokine threshold, and frequencies of cytokine-producing cells were calculated per cluster. All antigen-specific cytokine frequencies to PfRBCs are reported after uRBC subtraction, as a percentage of parents.

### Statistics.

All statistical analyses were performed using R software version 4.0.2 (36). The GLME model was used to assess the difference in cell clusters between the study groups and the changes in cell frequency after PfSPZ inoculation (37). Similarly, the GLME model was used to assess the difference at c–1 and the change after CHMI in the frequency of cytokine- producing cells per group. Specifically, to assess the change in cell frequencies over time, an interaction term among the groups variable and the time points variable was included. The model was computed using the GLMER package in R software. *P* values were adjusted for multiple comparisons using the FDR method. The significance level was set at *P* ≤ 0.05 after FDR correction. For the phenotyping assay, the frequency of each cell cluster as percentage of total cells (CD45^+^ cells) was determined. For cytokine production after stimulation, the frequency of cytokine-producing cells was determined as a percentage of their respective cluster. Using the R package survminer, we generated survival curves.

### Study approval.

The TüCHMI-002 trial was approved by the ethics committee of the Medical Faculty and the University Clinics of the University of Tübingen and registered at ClinicalTrials.gov (ClinicalTrials.gov NCT02115516) and in the EudraCT database (2013-003900-38). The trial followed the principles of the Declaration of Helsinki, Good Clinical Practice, and Good Clinical and Laboratory Practice. The study was carried out under FDA IND 15862 and with the approval of the Paul-Ehrlich-Institute (Langen, Germany).

### Data availability.

The mass cytometry data are available upon request from the corresponding author. Values for all data points in graphs are reported in the Supporting Data Values file.

## Author contributions

YDM designed the immunological study, conducted experiments, acquired data, analyzed the data using Cytosplore, performed the statistical analysis, and wrote the original draft of the manuscript. SPJ supervised the study, performed statistical analysis, and wrote the original draft of the manuscript. VVU analyzed the data using Cytosplore. MEBO conducted experiments. WAADSP and KAS analyzed data for the rebuttal and gave input on the statistical analysis. MML supervised the study. BKLS manufactured the inoculated product. SLH manufactured the inoculated product and designed the clinical trial. ME, PGK, and RF designed and conducted the clinical trial. BM designed and conducted the clinical trial and wrote the original draft of the manuscript. MY designed the immunological study, acquired funding, supervised the study, validated the data analysis, and wrote the original draft of the manuscript.

## Supplementary Material

Supplemental data

Supporting data values

## Figures and Tables

**Table 1:**
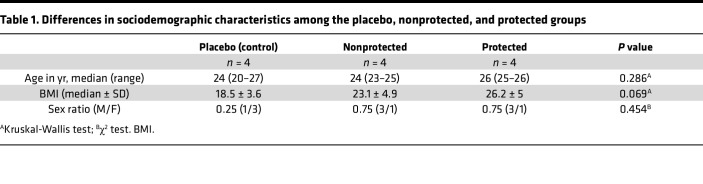
Differences in sociodemographic characteristics among the placebo, nonprotected, and protected groups

## Version 1 05/08/2024

Electronic publication

## References

[1] Verma R (2013). Malaria vaccine can prevent millions of deaths in the world. Hum Vaccin Immunother.

[2] https://www.who.int/publications/i/item/9789240086173.

[3] Datoo MS (2022). Efficacy and immunogenicity of R21/Matrix-M vaccine against clinical malaria after 2 years’ follow-up in children in Burkina Faso: a phase 1/2b randomised controlled trial. Lancet Infect Dis.

[4] Arora N (2021). Towards eradication of malaria: is the WHO’s RTS,S/AS01 vaccination effective enough?. Risk Manag Healthc Policy.

[5] RTS,S Clinical Trials Partnership. (2015). Efficacy and safety of RTS,S/AS01 malaria vaccine with or without a booster dose in infants and children in Africa: final results of a phase 3, individually randomised, controlled trial. Lancet.

[6] Mordmüller B (2017). Sterile protection against human malaria by chemoattenuated PfSPZ vaccine. Nature.

[7] Mwakingwe-Omari A (2021). Two chemoattenuated PfSPZ malaria vaccines induce sterile hepatic immunity. Nature.

[8] Sulyok Z (2021). Heterologous protection against malaria by a simple chemoattenuated PfSPZ vaccine regimen in a randomized trial. Nat Commun.

[9] Ishizuka AS (2016). Protection against malaria at 1 year and immune correlates following PfSPZ vaccination. Nat Med.

[10] Manurung MD (2022). Immunological profiles associated with distinct parasitemic states in volunteers undergoing malaria challenge in Gabon. Sci Rep.

[11] Epstein JE (2011). Live attenuated malaria vaccine designed to protect through hepatic CD8^+^ T cell immunity. Science.

[12] Weiss WR, Jiang CG (2012). Protective CD8+ T lymphocytes in primates immunized with malaria sporozoites. PLoS One.

[13] Bendall SC (2012). A deep profiler’s guide to cytometry. Trends Immunol.

[14] Reeves PM (2018). Application and utility of mass cytometry in vaccine development. FASEB J.

[15] De Jong SE (2021). Systems analysis and controlled malaria infection in Europeans and Africans elucidate naturally acquired immunity. Nat Immunol.

[16] Bandura DR (2009). Mass cytometry: technique for real time single cell multitarget immunoassay based on inductively coupled plasma time-of-flight mass spectrometry. Anal Chem.

[17] Van Unen V (2017). Visual analysis of mass cytometry data by hierarchical stochastic neighbour embedding reveals rare cell types. Nat Commun.

[18] Höllt T (2016). Cytosplore: interactive immune cell phenotyping for large single-cell datasets. Comput Graph Forum.

[19] Lell B (2018). Impact of sickle cell trait and naturally acquired immunity on uncomplicated malaria after controlled human malaria infection in adults in Gabon. Am J Trop Med Hyg.

[20] Watanabe H (2003). Expansion of unconventional T cells with natural killer markers in malaria patients. Parasitol Int.

[21] Jagannathan P (2017). Vδ2+ T cell response to malaria correlates with protection from infection but is attenuated with repeated exposure. Sci Rep.

[22] Van Braeckel-Budimir N, Harty JT (2014). CD8 T-cell-mediated protection against liver-stage malaria: lessons from a mouse model. Front Microbiol.

[23] Overstreet MG (2008). Protective CD8 T cells against Plasmodium liver stages: immunobiology of an ‘unnatural’ immune response. Immunol Rev.

[24] Schwenk R (2013). Ex vivo tetramer staining and cell surface phenotyping for early activation markers CD38 and HLA-DR to enumerate and characterize malaria antigen-specific CD8+ T-cells induced in human volunteers immunized with a Plasmodium falciparum adenovirus-vectored malaria vaccine expressing AMA1. Malar J.

[25] Noé A (2022). Deep immune phenotyping and single-cell transcriptomics allow identification of circulating TRM-like cells which correlate with liver-stage immunity and vaccine-induced protection from malaria. Front Immunol.

[26] Ewer KJ (2013). Protective CD8+ T-cell immunity to human malaria induced by chimpanzee adenovirus-MVA immunisation. Nat Commun.

[27] Chakravarty S (2007). CD8+ T lymphocytes protective against malaria liver stages are primed in skin-draining lymph nodes. Nat Med.

[28] Montoya CJ (2007). Characterization of human invariant natural killer T subsets in health and disease using a novel invariant natural killer T cell-clonotypic monoclonal antibody, 6B11. Immunology.

[29] Gonzalez-Aseguinolaza G (2000). alpha -galactosylceramide-activated Valpha 14 natural killer T cells mediate protection against murine malaria. Proc Natl Acad Sci U S A.

[30] Akondy RS (2009). The yellow fever virus vaccine induces a broad and polyfunctional human memory CD8+ T cell response. J Immunol.

[31] Minervina AA (2020). Primary and secondary anti-viral response captured by the dynamics and phenotype of individual T cell clones. Elife.

[32] Graham N (2020). Rapid induction and maintenance of virus-specific CD8^+^ T_EMRA_ and CD4^+^ T_EM_ cells following protective vaccination against dengue virus challenge in humans. Front Immunol.

[33] Geginat J (2003). Proliferation and differentiation potential of human CD8+ memory T-cell subsets in response to antigen or homeostatic cytokines. Blood.

[34] Callender LA (2018). Human CD8^+^ EMRA T cells display a senescence-associated secretory phenotype regulated by p38 MAPK. Aging Cell.

[35] Akondy RS (2017). Origin and differentiation of human memory CD8 T cells after vaccination. Nature.

[36] https://www.r-project.org/.

[37] Bates D (2015). Fitting linear mixed-effects models using lme4. J Stat Softw.

