# Supplementary material for: Immune responses associated with protection induced by chemoattenuated PfSPZ vaccine in malaria-naive Europeans
Source: JCI Insight. 2024 May 8;9(9):e170210. doi: 10.1172/jci.insight.170210 (PMC11141902; doi:10.1172/jci.insight.170210)
Supplement: Supplemental data [file jciinsight-9-170210-s260.pdf]

Supplementary Table 1: Lineage and subset labels for single live CD45^{+} cells

Lineage	Subset	Marker expression
CD4^{+} T cells	Naïve CD4^{+} T cells	CD3^{+} CD4^{+} CD8^{-} CD45RA^{+} CD45RO^{-} CCR7^{+}
	EM CD161^{+} CD4^{+} T cells	CD3^{+} CD4^{+} CD8^{-} CD45RA^{-} CD45RO^{+} CCR7^{-} CD161^{+}
	EM CD161^{-} CD4^{+} T cells	CD3^{+} CD4^{+} CD8^{-} CD45RA^{-} CD45RO^{+} CCR7^{-} CD161^{-}
	CM CD161^{+} CD4^{+} T cells	CD3^{+} CD4^{+} CD8^{-} CD45RA^{-} CD45RO^{+} CCR7^{+} CD161^{+}
	CM CD161^{-} CD4^{+} T cells	CD3^{+} CD4^{+} CD8^{-} CD45RA^{-} CD45RO^{+} CCR7^{-} CD161^{-}
	CD25^{+} CD4^{+} T cells	CD3^{+} CD4^{+} CD8^{-} CD25^{+}
	CD56^{+} CD4^{+} T cells	CD3^{+} CD4^{+} CD8^{-} CD45RA^{+} CD45RO^{-} or CD45RO^{+}
CD8^{+} T cells	Naïve CD8^{+} T cells	CD3^{+} CD4^{-} CD8^{+} CD45RA^{+} CD45RO^{-} CCR7^{+}
	EM CD8 T cells	CD3^{+} CD4^{-} CD8^{+} CD45RA^{-} CD45RO^{+} CCR7^{-}
	CM CD8 T cells	CD3^{+} CD4^{-} CD8^{+} CD45RA^{-} CD45RO^{+} CCR7^{+}
	CD56^{+} CD8 T cells	CD3^{+} CD4^{-} CD8^{+} CD45RA^{+} CD45RO^{-} or CD45RO^{+}
	EMRA CD8 T cells	CD3^{+} CD4^{-} CD8^{+} CD45RA^{+} CD45RO^{-} CCR7^{-}
γδ T cells	CD161^{+} γδ T cells	CD3^{+} TCRγδ^{+} CD161^{+}
	CD56^{+} γδ T cells	CD3^{+} TCRγδ $^{+}$ CD56^{+}
	CD45RA or CD45RO γδ T cells	CD3^{+} TCRγδ^{+} CD45RA^{+} CD45RO^{-} or CD45RA^{-} CD45RO^{+}
Unconventional T cells	DN T cells	CD3^{+} CD4^{-} CD8^{-}
	CD161hi CD8lo T cells	CD3^{+} CD4^{-} CD8^{lo} CD161^{hi}
B cells	IgD^{-} or IgD^{+} B cells	CD19^{+} IgD^{-} or IgD^{+}
	CD11c^{+} Bcells	CD19^{+} CD11c^{+}
	Plasma cells	CD19^{+} CD27^{hi} CD38^{+}
ILCs	NK cells	CD3^{-} CD19^{-} CD14^{-} CD56^{+} and/or CD16^{+}
	ILC2s	CD3^{-} CD19^{-} CD14^{-} CD25^{+} CD117^{-} CD161^{+} CRTH2^{+}
	ILC3s	CD3^{-} CD19^{-} CD14^{-} CD25^{+} CD117^{+} CD161^{+} CRTH2^{-}
Monocytes and DCs	Classical monocytes	CD11c^{+} HLA-DR^{+} CD14^{+} CD16^{-}
	non-classical and intermediate monocytes	CD11c^{+} HLA-DR^{+} CD14^{+} CD16^{+} or CD14^{-} CD16^{+}
	pDCs	HLA-DR^{+} CD123^{+} CD4^{low}
Basophils	Basophils	HLA-DR^{-} CD123^{+} CD25^{+} CRTH2^{+} CD38^{+}

Supplementary Table 2: Surface panel.

Label	Marker	Clone	Company	Catalogue Number	dilution
**^{89}Y**	CD45	HI30	Fluidijm[a]	3089003B	200
**^{113}CD**	CD45RA	HI100	eBiosciences[b]	83-0458-42	50
**^{141}Pr**	CD196 (CCR6)	G034E3	Fluidijm	3141003A	100
**^{142}Nd**	CD19	HIB19	Fluidijm	3142001B	200
**^{143}Nd**	CD117 (c-Kit)	104D2	Fluidijm	3143001B	100
**^{145}Nd**	CD4	RPA-T4	Fluidijm	3145001B	100
**^{146}Nd**	CD8a	RPA-T8	Fluidijm	3146001B	200
**^{147}Sm**	CD183 (CXCR3)	G025H7	Biolegend[c]	353733	100
**^{148}Nd**	CD14	M5E2	Biolegend	301843	100
**^{149}Sm**	CD25 (IL-2Ra)	2A3	Fluidijm	3149010B	100
**^{150}Nd**	CD185 (CXCR5)	J252D4	Biolegend	356902	100
**^{151}Eu**	CD123	6H6	Biolegend	313502	100
**^{152}Sm**	TCRγδ	11F2	Fluidijm	3152008B	50
**^{153}Eu**	CD7	CD7-6B7	Fluidijm	3153014B	100
**^{154}Sm**	CD163	GHI/61	Fluidijm	3154007B	100
**^{155}Gd**	CD69	FN50	Biolegend	310939	200
**^{156}Gd**	CD294 (CRTH2)	BM16	Biolegend	350102	50
**^{158}Gd**	CD122 (IL-2Rb)	TU27	Biolegend	339015	100
**^{159}Tb**	CD197 (CCR7)	G043H7	Fluidijm	3159003A	100
**^{161}Dy**	KLRG1 (MAFA)	REA261	Miltenyi[d]	Special order	100
**^{162}Dy**	CD11c	Bu15	Fluidijm	3162005B	200
**^{163}Dy**	IgD	IA6-2	Biolegend	348235	200
**^{164}Dy**	CD161	HP-3G10	Fluidijm	3164009B	100
**^{165}Ho**	CD127 (IL-7Ra)	AO19D5	Fluidijm	3165008B	200
**^{166}Er**	CD141 (BDCA3)	1A4	BD Biosciences[e]	559780	200
**^{167}Er**	CD27	O323	Fluidijm	3167002B	200
**^{168}Er**	HLA-DR	L243	Biolegend	307651	200
**^{170}Er**	CD3	UCHT1	Fluidijm	3170001B	100
**^{171}Yb**	CD28	CD28.2	Biolegend	302937	200
**^{172}Yb**	CD38	HIT2	Fluidijm	3172007B	200
**^{173}Yb**	CD45RO	UCHL1	Biolegend	304239	100
**^{174}Yb**	CD335 (NKp46)	92E	Biolegend	331908	100
**^{175}Lu**	CD279 (PD-1)	EH 12.2H7	Fluidijm	3175008B	100
**^{176}Yb**	CD56	NCAM16.2	Fluidijm	3176008B	100
**^{198}Pt**	CD86	IT2.2	Biolegend	305435	200
**^{209}BI**	CD16	3G8	Fluidijm	3209002B	400

[a]Fluidigm, South San Francisco, CA, USA. [b]eBioscience, San Diego, CA, USA. [c]Biolegend, San Diego, CA, USA. [d]Miltenyi Biotech, Bergisch Gladbach, Germany. [d]BD Biosciences, San Jose, CA, USA. CCR, C-C chemokine receptor. CD, cluster of differentiation. CRTH2, prostaglandin D2 receptor 2. CXCR, CXC chemokine receptor. HLA-DR, human leukocyte antigen-D-related. IL-2R, interleukin-2 receptor. IL7Rα, interleulin- 7 receptor α. KLRG1, killer cell lectin-like receptor subfamily G member 1. MAFA, mast cell function-associated antigen. PD-1, programmed cell death protein. TCR, T-cell receptor.

Supplementary Table 3: Cytokine panel.

Label	Marker	Clone	Company	Catalogue Number	dilution
**089Y**	CD45	HI30	Fluidijm[a]	3089003B	200
**113CD**	CD45RA	HI100	eBiosciences[b]	83-0458-42	50
**141Pr**	CD196 (CCR6)	G034E3	Fluidijm	3141003A	100
**142Nd**	CD19	HIB19	Fluidijm	3142001B	200
**143Nd**	CD117 (c-Kit)	104D2	Fluidijm	3143001B	100
**144Nd**	IL2	MQ117H12	Biolegend[c]	500339	400
**145Nd**	CD4	RPA-T4	Fluidijm	3145001B	100
**146Nd**	CD8a	RPA-T8	Fluidijm	3146001B	200
**147Sm**	CD183 (CXCR3)	G025H7	Biolegend	353733	100
**148Nd**	CD14	M5E2	Biolegend	301843	100
**149Sm**	CD25 (IL-2Ra)	2A3	Fluidijm	3149010B	100
**150Nd**	CD185 (CXCR5)	J252D4	Biolegend	356902	100
**151Eu**	CD123	6H6	Biolegend	313502	100
**152Sm**	TCRγδ	11F2	Fluidijm	3152008B	50
**153Eu**	CD7	CD7-6B7	Fluidijm	3153014B	100
**154Sm**	CD163	GHI/61	Fluidijm	3154007B	100
**155Gd**	IFNg	B27	Biolegend	506521	400
**156Gd**	CD294 (CRTH2)	BM16	Biolegend	350102	50
**158Gd**	CD122 (IL-2Rb)	TU27	Biolegend	339015	100
**159Tb**	CD197 (CCR7)	G043H7	Fluidijm	3159003A	100
**160Gd**	TNFa	MAb11	Biolegend	502941	400
**161Dy**	KLRG1 (MAFA)	REA261	Miltenyi[d]	Special order	100
**162Dy**	CD11c	Bu15	Fluidijm	3162005B	200
**163Dy**	IL-17	BL168	Biolegend	512331	400
**164Dy**	CD161	HP-3G10	Fluidijm	3164009B	100
**165Ho**	CD127 (IL-7Ra)	AO19D5	Fluidijm	3165008B	200
**166Er**	IL10	JES39D7	Fluidijm	3166008B	400
**167Er**	CD27	O323	Fluidijm	3167002B	200
**168Er**	HLA-DR	L243	Biolegend	307651	200
**169Tm**	IL4	MP4-25D2	Fluidijm	3169016B	400
**169Tm**	IL5	TRFK5	Biolegend	500829	400
**169Tm**	IL13	JES105A2	Biolegend	504309	400
**170Er**	CD3	UCHT1	Fluidijm	3170001B	100
**171Yb**	CD28	CD28.2	Biolegend	302937	200
**172Yb**	CD38	HIT2	Fluidijm	3172007B	200
**173Yb**	CD45RO	UCHL1	Biolegend	304239	100
**174Yb**	CD335 (NKp46)	92E	Biolegend	331908	100
**175Lu**	CD279 (PD-1)	EH 12.2H7	Fluidijm	3175008B	100
**176Yb**	CD56	NCAM16.2	Fluidijm	3176008B	100
**198Pt**	CD86	IT2.2	Biolegend	305435	200
**209BI**	CD16	3G8	Fluidijm	3209002B	400

[a]Fluidigm, South San Francisco, CA, USA. [b]eBioscience, San Diego, CA, USA. [c]Biolegend, San Diego, CA, USA. [d]Miltenyi Biotech, Bergisch Gladbach, Germany. CCR, C-C chemokine receptor. CD, cluster of differentiation. CRTH2, prostaglandin D2 receptor 2. CXCR, CXC chemokine receptor. HLA-DR, human

leukocyte antigen-D-related. IL-2R, interleukin-2 receptor. IL7Rα, interleulin- 7 receptor α. KLRG1, killer cell lectin-like receptor subfamily G member 1. MAFA, mast cell function-associated antigen. PD-1, programmed cell death protein. TCR, T-cell receptor. Markers in grey were stained intracellular, while all other markers were stained on the cell surface.

Supplementary Table 4: Parasites per mL of blood, assessed by qPCR and by thick blood smear (TBS), per donor within the different study groups. Daily analyses were performed from day 6 to 21 post-inoculation of PfSPZ. Slides were considered positive when at least two parasites were detected within 300 microscope fields of view. If only one parasite-like structure was identified in 300 fields by a microscopist the slide was reported as negative to avoid false-positive results because of staining artefacts. Two trained microscopists examined each slide. In case of discrepancy between the results of the two readers, a third expert reader assessed the slide. The day of TBS positivity correlates with the peak of parasitemia detected by qPCR. After positive results from both PCR and TBS, further assessment of parasitemia was not conducted. Therefore, those particular days are indicated as N/A (not applicable) in the table.

			Day of parasitemia test															
**Participant ID**	**Group**	**Parasitemia (parasite/mL)**	**day 6**	**day 7**	**day 8**	**day 9**	**day 10**	**day 11**	**day 12**	**day 13**	**day 14**	**day 15**	**day 16**	**day 17**	**day 18**	**day 19**	**day 20**	**day 21**
**T2.002**	Protected	qPCR	0	0	0	0	0	0	0	0	0	0	0	0	0	0	0	0
**T2.006**	Protected	qPCR	0	0	0	0	0	0	0	0	0	0	0	0	0	0	0	0
**T2.038**	Protected	qPCR	0	0	0	0	0	0	0	0	0	0	0	0	0	0	0	0
**T2.067**	Protected	qPCR	0	0	0	0	0	0	0	0	0	0	0	0	0	0	0	0
**T2.010**	Non-Protected	qPCR	0	2	26	20	0	2059	4066	7185	N/A	N/A	N/A	N/A	N/A	N/A	N/A	N/A
**T2.012**	Non-Protected	qPCR	0	106	71	1150	933	10501	44300	N/A	N/A	N/A	N/A	N/A	N/A	N/A	N/A	N/A
**T2.022**	Non-Protected	qPCR	0	11	99	295	171	7521	5494	83570	N/A	N/A	N/A	N/A	N/A	N/A	N/A	N/A
**T2.046**	Non-Protected	qPCR	0	6	12	52	52	373	2393	792	N/A	N/A	N/A	N/A	N/A	N/A	N/A	N/A
**T2.005**	Placebo	qPCR	0	108	1077	909	482	14376	N/A	N/A	N/A	N/A	N/A	N/A	N/A	N/A	N/A	N/A
**T2.047**	Placebo	qPCR	0	7	903	299	414	2213	N/A	N/A	N/A	N/A	N/A	N/A	N/A	N/A	N/A	N/A
**T2.055**	Placebo	qPCR	0	57	626	381	1009	12048	20202	N/A	N/A	N/A	N/A	N/A	N/A	N/A	N/A	N/A
**T2.063**	Placebo	qPCR	0	39	112	27	2	43	1	34	74	1288	550	2399	48978	N/A	N/A	N/A
**T2.002**	Protected	TBS	0	0	0	0	0	0	0	0	0	0	0	0	0	0	0	0
**T2.006**	Protected	TBS	0	0	0	0	0	0	0	0	0	0	0	0	0	0	0	0
**T2.038**	Protected	TBS	0	0	0	0	0	0	0	0	0	0	0	0	0	0	0	0
**T2.067**	Protected	TBS	0	0	0	0	0	0	0	0	0	0	0	0	0	0	0	0
**T2.010**	Non-Protected	TBS	0	0	0	0	0	0	0	1	N/A	N/A	N/A	N/A	N/A	N/A	N/A	N/A
**T2.012**	Non-Protected	TBS	0	0	0	0	0	0	7		N/A	N/A	N/A	N/A	N/A	N/A	N/A	N/A
**T2.022**	Non-Protected	TBS	0	0	0	0	0	0	0	12	N/A	N/A	N/A	N/A	N/A	N/A	N/A	N/A
**T2.046**	Non-Protected	TBS	0	0	0	0	0	0	0	1	N/A	N/A	N/A	N/A	N/A	N/A	N/A	N/A
**T2.005**	Placebo	TBS	0	0	0	0	0	4	N/A	N/A	N/A	N/A	N/A	N/A	N/A	N/A	N/A	N/A
**T2.047**	Placebo	TBS	0	0	0	0	0	4	N/A	N/A	N/A	N/A	N/A	N/A	N/A	N/A	N/A	N/A
**T2.055**	Placebo	TBS	0	0	0	0	0	0	3	N/A	N/A	N/A	N/A	N/A	N/A	N/A	N/A	N/A
**T2.063**	Placebo	TBS	0	0	0	0	0	0	0	0	0	0	0	0	1	N/A	N/A	N/A

<u>Supplementary Table 5</u>: Change in cluster frequencies from c-1 to d11 per group (Placebo, Non-protected and Protected). The change overtime was analyzed per group using the linear mixed model, and the p-value was adjusted using false discovery rate (FDR) correction. In red are the significant p-values ($p \leq 0.05$) per clusters after FDR correction within each group.

		FDR_ p-Value (c-1 to d-11)		
**Lineage**	**Cluster_ID**	**Placebo**	**Non-protected**	**Protected**
**CD4**	3	0,0010	0,8752	0,7483
**CD4**	4	0,0007	0,6320	0,8556
**CD4**	6	0,0382	0,7610	0,9307
**CD4**	9	0,0236	0,0421	0,8556
**CD4**	13	0,0041	0,6831	0,9307
**CD4**	22	0,4674	0,7676	<0.0001
**CD4**	24	0,0505	0,6467	0,8556
**CD4**	27	0,5403	0,0990	<0.0001
**CD4**	35	<0.0001	0,8345	0,7483
**CD4**	36	0,2208	0,7610	0,0531
**CD4**	37	0,7865	0,6320	0,0011
**CD4**	38	0,0367	0,0390	0,9490
**CD4**	40	0,0137	0,8188	0,9307
**CD4**	41	0,0576	0,8752	0,9534
**CD8**	44	0,3322	0,7610	0,0017
**CD8**	46	0,9875	0,9553	<0.0001
**CD8**	49	0,5020	0,7676	0,0468
**CD8**	50	0,0599	0,8464	0,9307
**CD8**	51	0,0185	0,7610	0,8556
**CD8**	55	0,0007	0,6320	0,4651
**CD8**	63	0,0505	0,8752	0,8247
**CD8**	64	0,0382	0,8908	0,8556
**CD8**	67	0,2293	0,9052	0.001
**CD8**	71	<0.0001	0,9394	0,9307
**CD8**	74	<0.0001	0,8752	0,7612
**γδT**	76	0,0185	0,9790	0,8556
**γδT**	81	0,7326	0,8752	0,0011
**CD8**	93	<0.0001	0,0421	0
**CD8**	96	0,6938	0,7610	<0.0001
**Unconventional t cells**	102	0,0576	0,8464	0,8556
**Unconventional t cells**	105	0,8427	0,0160	0,8556
**Unconventional t cells**	107	<0.0001	0,8561	0,7821
**B**	116	<0.0001	0,6831	0,9490
**B**	119	0,0505	0,7923	0,9490
**B**	120	0,0392	0,5850	0,8556
**B**	121	0,8527	0,6320	0,0011
**ILCs**	122	0,6739	0,8908	0,0188
**ILCs**	123	0,0198	0,7610	0,8556
**ILCs**	130	0,2002	0,9790	<0.0001
**Monocytes and DCs**	131	0,0505	0,6831	0,8556
**Monocytes and DCs**	135	0,0137	0,8752	0,8556
**Monocytes and DCs**	138	0,0726	0,7610	<0.0001
**Monocytes and DCs**	139	<0.0001	0,7610	0,0059

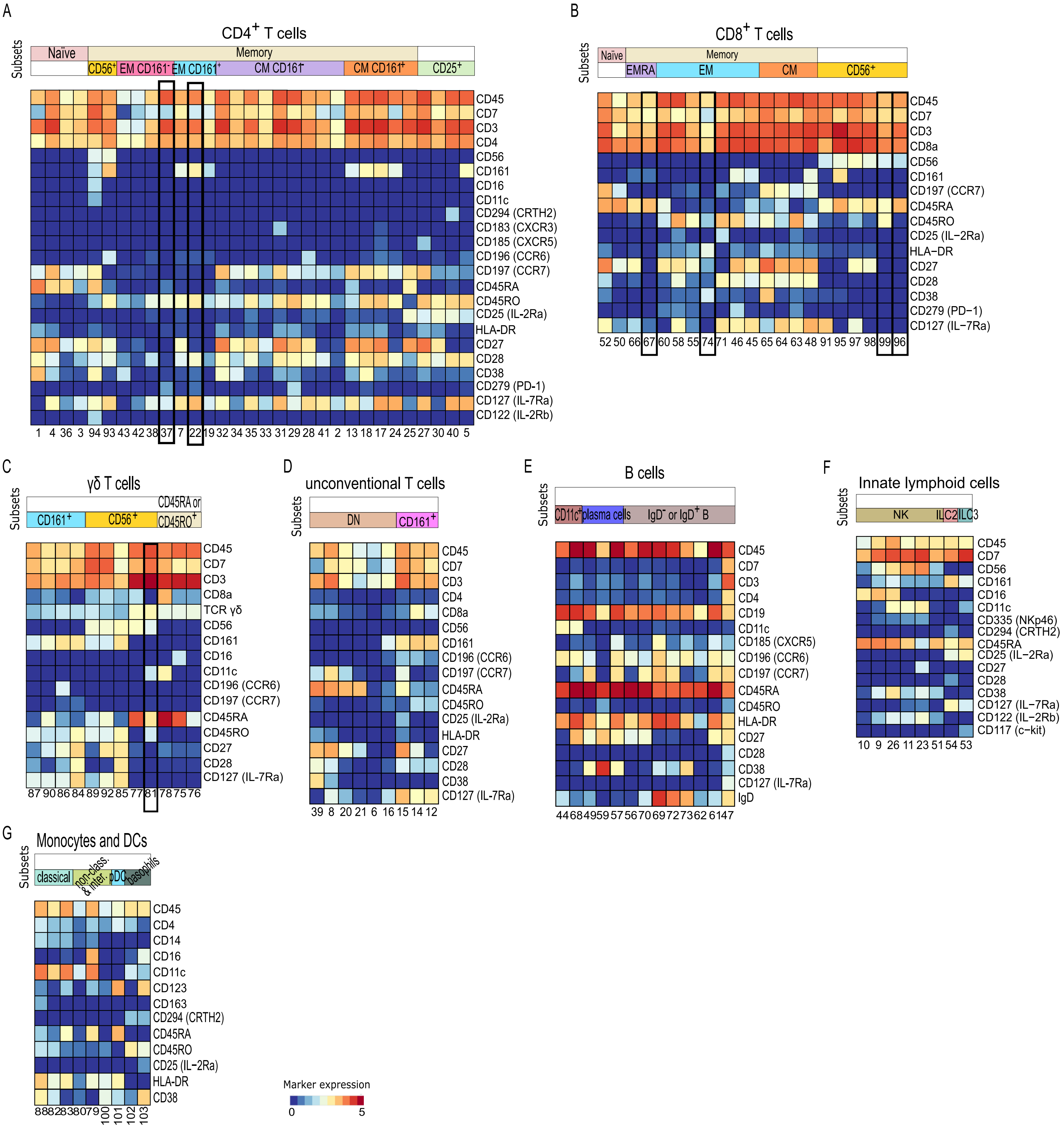

Supplementary Figure 1: Heatmap showing expression of markers as median signal intensity (MSI) after arsinh transformation for 103 clusters identified. Each cluster has a unique cluster number and are shown within (A) CD4$^+$ T cells, (B) CD8$^+$ T cells, (C) γδ T cell, (D) unconventional T cells, (E) B cells, (F) innate lymphoid cells, and (G) monocytes and DCs lineages. Subsets to which each cluster belongs is shown in the upper horizontal panels. The colors are specific per row and lineages and are independent of colors in others rows and lineages

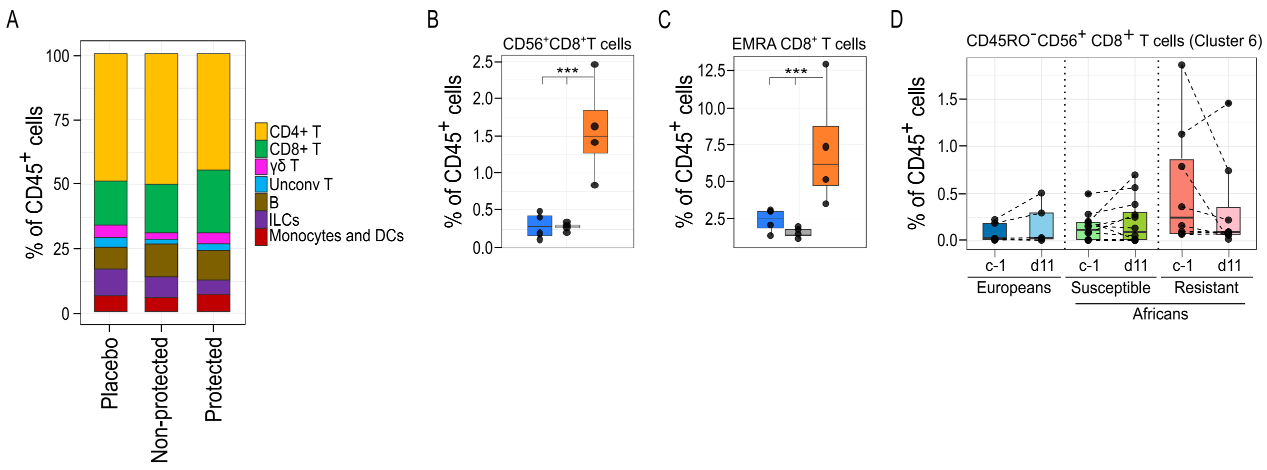

Supplementary Figure 2: (A) Cell frequency relative to total CD45$^+$ cells between placebo, non-protected, and protected per lineages. Differences between the groups in lineages abundance were tested using the GLME model. (B) CD56$^+$ CD8$^+$ T cells subset frequency relative to CD45$^+$ cells compared between placebo (n= 4) in blue, non-protected (n= 4) in grey, and protected (n= 4) in orange; FDR-adjusted p-value <0.001. (C) EMRA CD8$^+$ T cells subset frequency relative to CD45$^+$ cells between groups as indicated; FDR - adjusted p-value< 0.001. (D) CD56$^+$ CD8$^+$ T cell cluster negative for CD45RO expression (cluster 6) from c-1 to d11, as percentage of CD45$^+$, between Europeans (n= 5), susceptible (n= 12) and resistant Africans (n= 8). Data in B, C, and D are representing in boxplots showing the median, the 1st and 3rd quartile, with the whiskers extended no further than 1.5x the interquartile range (IQR).

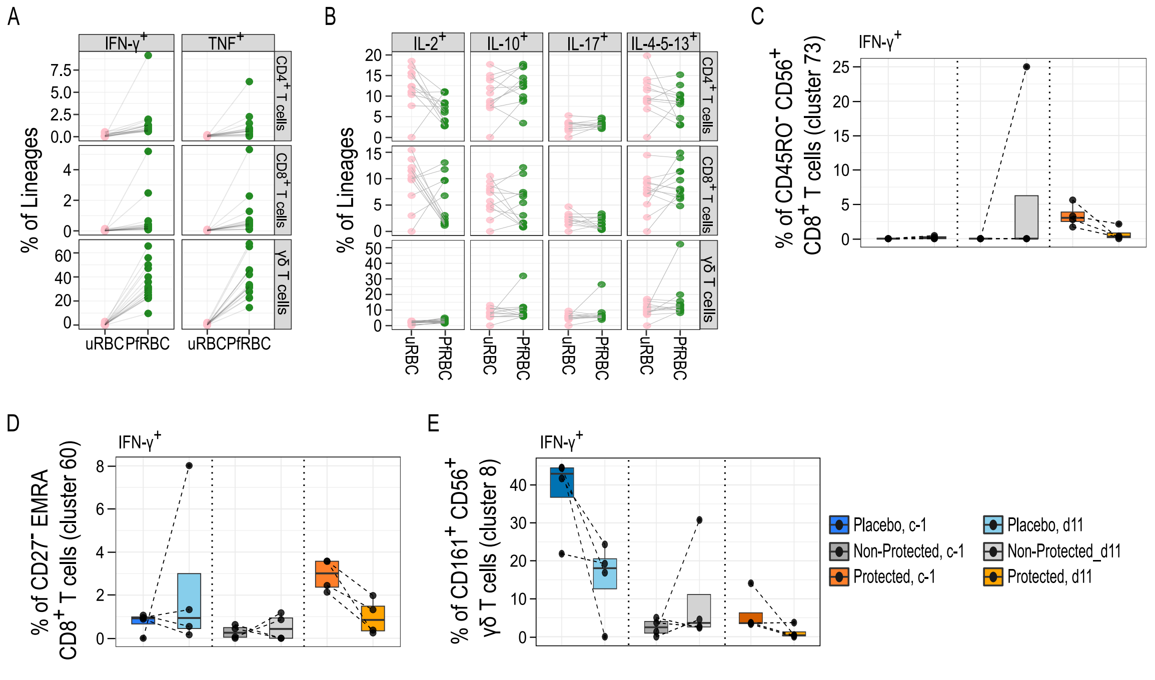

Supplementary Figure 3: (A) IFN-γ, TNF (B), IL-2, IL-10, IL-17, and IL-4-5-13 producing $CD4^+$, $CD8^+$ and γδ T cells between uRBC controls (pink) and PfRBC (green). (C) Frequency of IFN-γ producing $CD56^+$ $CD8^+$ T cells (cluster 73), (D) EMRA $CD8^+$ T cells (cluster 60), and (E) $CD56^+$ γδ T (cluster 8), from c-1 to d11 as percentage of parent, between the indicated groups. The boxplots represent the median, the 1st and the 3rd quartile, while the whiskers is extended no further than 1.5x the interquartile range (IQR).

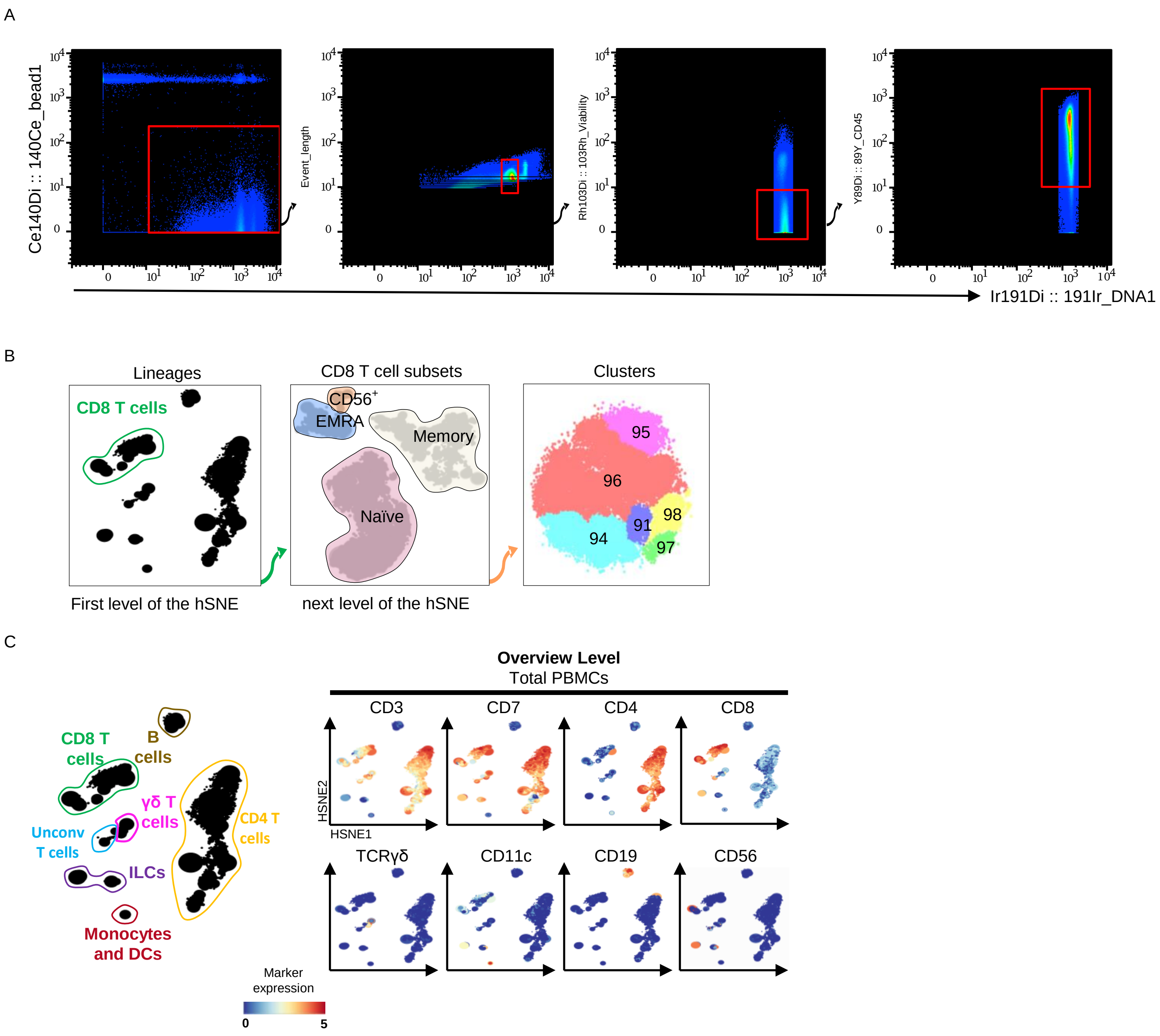

Supplementary Figure 4: (A) Flow cytometry gating strategy for CD45^{+} cells after Helios^{TM} analysis (Fluidigm). Using Flowjo software version 10, cells were first gated on beads and DNA to discriminate the cells from the calibration beads. Next, singlets were selected using even length and DNA. Thereafter, cells were gated using intercalator-103Rh and DNA to select live cells. Subsequently, CD45^{+} cells were gated. (B) HSNE analysis on Cytosplore. At the first level of the HSNE lineages are defined. At the next level, subsets are defined and so on until the cluster level is reached. The illustration here of CD56^{+} CD8^{+} T cells analysis in Cytosplore. (C) First HSNE level embedding depicting the major immune lineages.
